# 

**Published:** 1884-12

**Authors:** 


DECEMBER, 1884.

### THE AMERICAN JOURNAL OF DENTAL SCIENCE.

PUBLISHED MONTHLY.

$2.50 PER ANNUM.

EDITED BY

F. J. S. GORGAS, M. D., D. D. S.

Vol. 18. THIRD SERIES. No. 8

BALTIMORE:

SNOWDEN & COWMAN.

LONDON:

TRUBNER & CO., 60 PATERNOSTER ROW.

1883.

PAYABLE IN ADVANCE.

